# Renal Hydatid Cyst: Diagnostic Quandaries and Surgical Strategies

**DOI:** 10.7759/cureus.66289

**Published:** 2024-08-06

**Authors:** Pratik Taur, Deerush Kannan, Nandyala Penchala Reddy, Deepak Raghavan

**Affiliations:** 1 Urology, Apollo Hospitals, Chennai, IND

**Keywords:** complex hydatid cyst, bosniak 3/4 cyst, renal cyst, renal hydatid cyst, cyst hydatid

## Abstract

Renal hydatid cyst, an uncommon manifestation of *Echinococcus granulosus* infection, presents a diagnostic challenge due to its asymptomatic nature. Here, we report the case of a 34-year-old male who presented with intermittent left flank pain, abdominal fullness, and passage of whitish clots in urine. Physical examination revealed a palpable 15 cm × 11 cm hard mass extending from the left hypochondrium to the left lumbar region. Ultrasonography and contrast-enhanced computed tomography identified a 15 cm Bosniak type 3 complex cystic lesion arising from the left kidney, causing hydronephrosis and hydroureter. The patient underwent a left nephrectomy, and a histopathological examination confirmed a renal hydatid cyst. This case highlights the diagnostic difficulty in differentiating renal hydatid cysts from other renal lesions. Despite suggestive radiological findings, conclusive diagnosis remains elusive, particularly in solitary complex renal cysts. Awareness of renal hydatid cysts in the differential diagnosis is crucial for appropriate management.

## Introduction

Renal hydatid cyst is an infrequent pathology attributed to the larval stage of *Echinococcus granulosus* [1]. While hydatid cysts commonly afflict organs such as the liver and lung, occurrences in atypical locations including the heart, breast, thyroid, soft tissues of the neck, and kidneys are rare [1]. The renal manifestation of hydatid cysts is particularly elusive, often exhibiting an asymptomatic course for extended periods [2]. Symptomatic presentations typically entail flank pain and the presence of a palpable lump [2]. However, the potential rupture of large cysts can incite a vigorous immunological reaction, potentially culminating in urticaria and even anaphylaxis [1,2]. Moreover, cyst rupture into the renal collecting system can precipitate renal colic and hydatiduria [1,2]. The complexity of diagnosis was exemplified in a recent case encountered by our team, thereby underscoring the exigency for heightened clinical vigilance in approaching such clinical dilemmas.

## Case presentation

**Figure 1:**
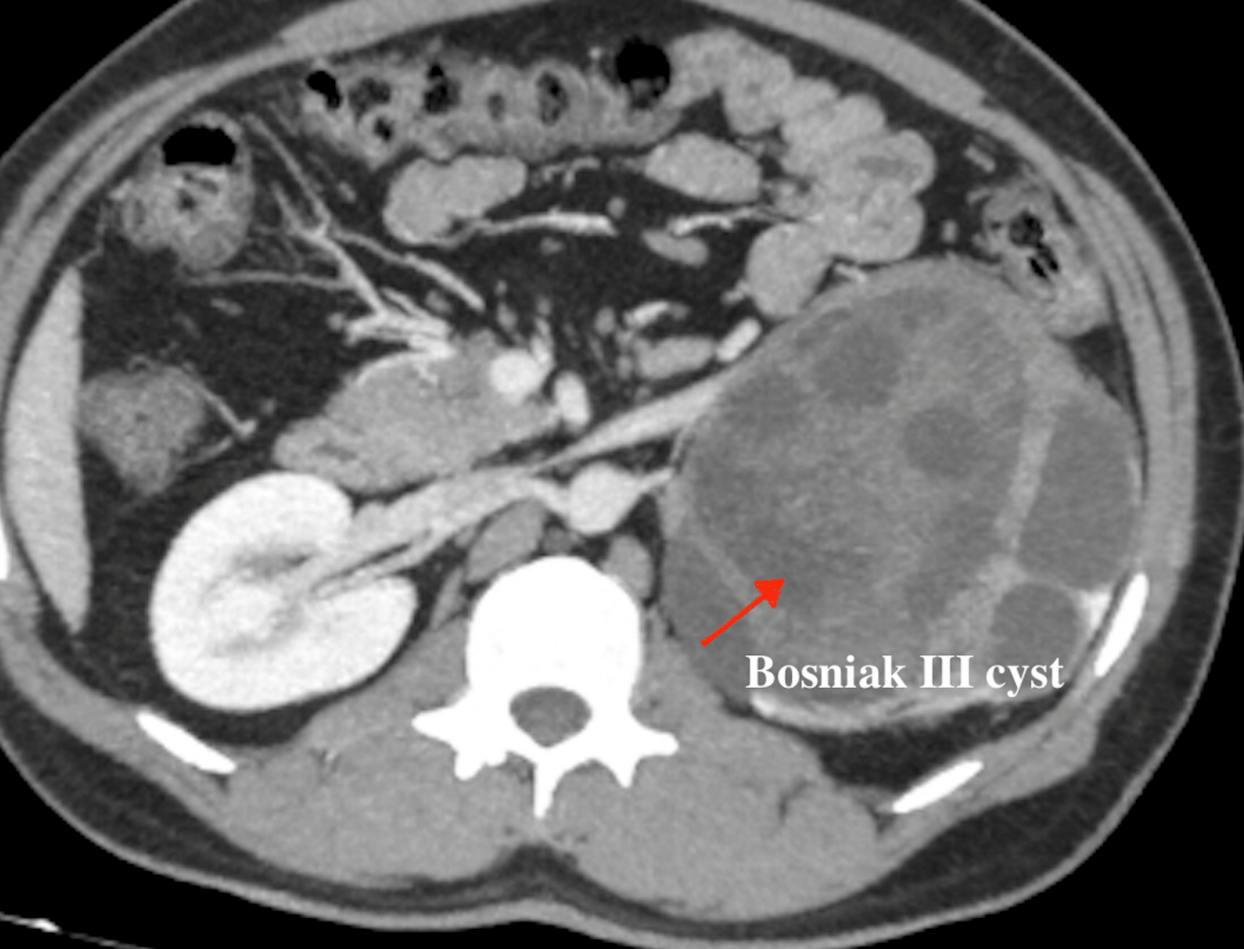
Bosniak type 3 cyst in the left kidney on the computed tomography scan.

**Figure 2:**
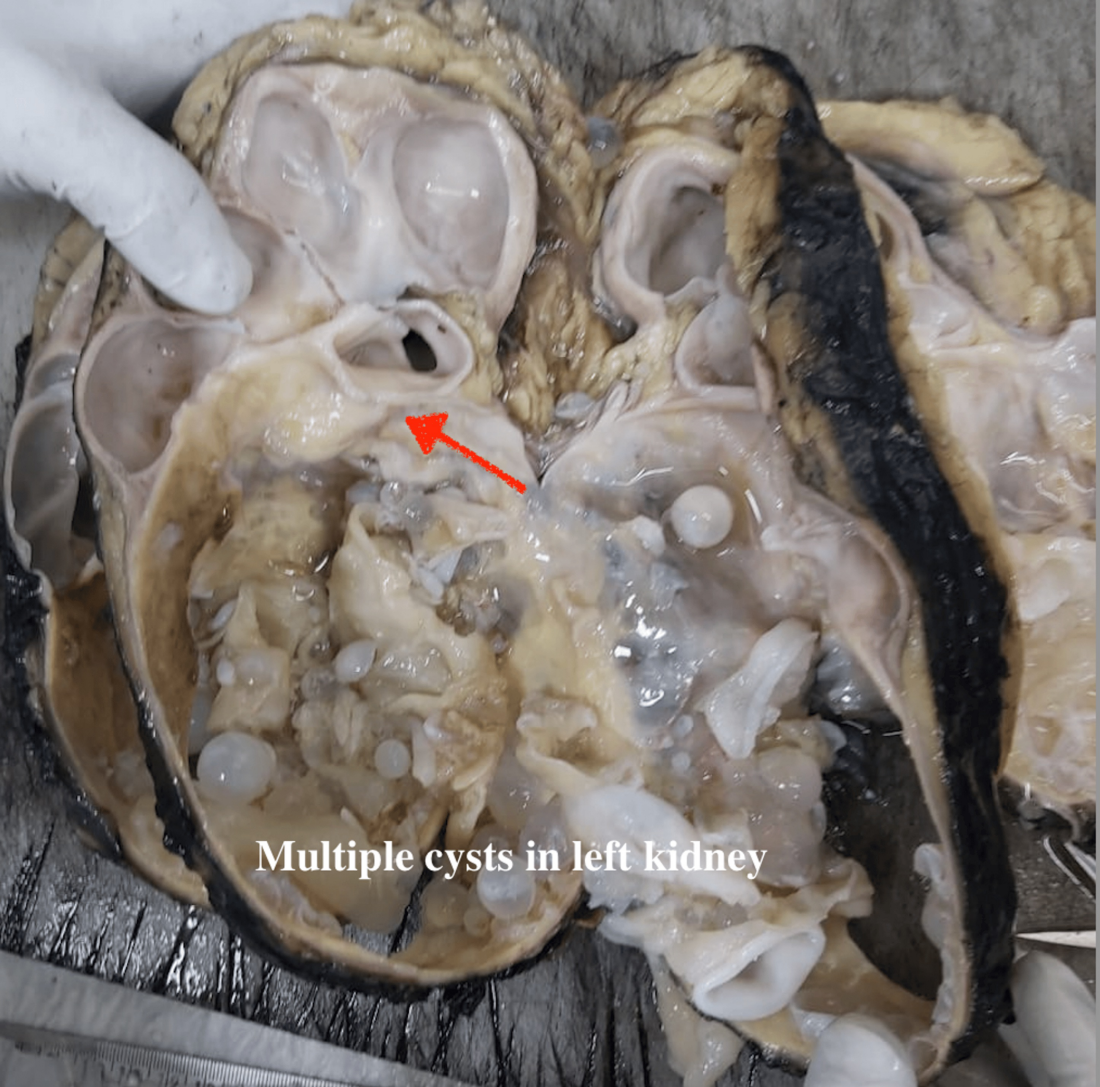
Gross image after the left nephrectomy showing multiple cysts in the kidney.

A 34-year-old Indian man with no positive medical history presented with intermittent left flank pain, abdominal fullness, and passage of turbid whitish clots in urine over a six-month period. He did not have any contact with any animals in the recent past. On physical examination, a palpable 15 cm × 11 cm × 10 cm hard mass extending from the left hypochondrium to the left lumbar region was noted. Ultrasonography of the kidneys, ureters, and bladder revealed a 15 cm solid cystic mass originating from the left kidney. Subsequent contrast-enhanced computed tomography of the kidneys, ureters, and bladder confirmed left hydronephrosis and hydroureter secondary to pelviureteric junction obstruction with thinned-out parenchyma due to a 15 cm Bosniak type 3 complex cystic lesion with a non-enhancing solid component (Figure 1). Preoperative blood parameters were within normal limits. Urine analysis showed no evidence of hematuria or hydaturia. The provisional diagnosis was a renal cystic tumor, and the patient underwent laparoscopically converted open left nephrectomy (Figure 2). Intraoperatively, dense adhesions and neovascularity were observed between the renal lesion and surrounding tissues. Given the large size and dense adhesions, the procedure was converted to open. The postoperative period was uneventful, and histopathological examination confirmed the presence of a renal hydatid cyst.

## Discussion

Isolated renal hydatid cyst is a rare entity, and reported cases in India, despite its endemic status for *Echinococcus*, are scarce. Renal involvement occurs in only 2-3% of hydatid disease cases [2]. The cyst typically presents as a uni- or multilocular well-defined lesion, with or without calcification, originating from the upper or lower pole of the kidney. These characteristics can often mimic other renal lesions such as complex cysts, multicystic nephroma, and cystic renal cell carcinoma [3]. Diagnosis of renal hydatid cysts poses a significant challenge, as radiological findings are suggestive but frequently inconclusive, often resembling renal complex cysts [4]. Imaging modalities may aid in confirming the diagnosis by revealing characteristic features such as layers, laminated membranes, or protoscolices within the hydatid cyst. While imaging plays a crucial role in diagnosis, serology has limited utility, primarily serving a confirmatory function due to the high rates of false-negative results [4].

The diagnostic ambiguity surrounding renal hydatid cysts complicates the evaluation of solitary complex renal cysts. Hence, renal hydatid cysts should be included in the differential diagnosis of such lesions. Surgical intervention carries the risk of fatal complications such as anaphylaxis and dissemination, emphasizing the importance of meticulous dissection to remove the cyst intact [5]. This necessitates complete mobilization of the kidney from surrounding tissues and cautious enucleation of the cyst without compromising the cyst wall or inadvertently suctioning its contents. These precautions are vital to prevent spillage of cyst contents and subsequent dissemination. In cases where preservation of renal function is paramount, excision with a healthy margin of kidney tissue, akin to partial nephrectomy, can be pursued for hydatid cyst removal.

The rarity and diagnostic challenges of renal hydatid cysts are established in the literature, where similar cases highlight the necessity of considering hydatid cysts in the differential diagnosis of complex renal lesions. A study by Polat et al. underscored the importance of advanced imaging techniques in differentiating hydatid cysts from other renal pathologies, emphasizing that features such as the presence of daughter cysts and detachment of the membrane can be indicative of hydatid disease [6]. Additionally, a review by Turgut et al. discussed the critical role of preoperative diagnosis in avoiding complications, reiterating that careful surgical planning is essential to prevent intraoperative spillage and anaphylactic reactions [7]. These findings reinforce the significance of including hydatid cysts in diagnostic considerations and employing meticulous surgical techniques to ensure patient safety and successful outcomes.

## Conclusions

In summary, diagnosing renal hydatid cysts remains challenging due to overlapping imaging features with other renal lesions. Despite suggestive radiological findings, definitive diagnosis often eludes clinicians, particularly in cases of solitary complex renal cysts. Therefore, renal hydatid cysts should be considered in the differential diagnosis of such lesions to ensure timely and appropriate management.
